# High Rates of Miscarriage and Stillbirth among Pregnant Women with Clade I Mpox (Monkeypox) Are Confirmed during 2023–2024 DR Congo Outbreak in South Kivu Province

**DOI:** 10.3390/v16071123

**Published:** 2024-07-13

**Authors:** David A. Schwartz

**Affiliations:** Perinatal Pathology Consulting, Atlanta, GA 30342, USA; davidalanschwartz@gmail.com

**Keywords:** monkeypox, mpox, monkeypox virus, DR Congo, miscarriage, stillbirth, pregnancy, placenta, congenital infection, spontaneous abortion, poxvirus, vertical transmission, fetal infection

## Abstract

Mpox (monkeypox) is a neglected tropical disease that has received increased attention since the multi-nation outbreak that began in 2022. The virus is endemic in West and Central Africa, where the Democratic Republic of the Congo (DRC) is the most affected country. Clade I monkeypox virus (MPXV) infection is endemic in the DRC and has an overall case fatality rate of 10.6% among children and adults. A study conducted in Sankuru Province, DRC, from 2007 to 2011 demonstrated that 75% of pregnant women with mpox had miscarriages or stillbirth. Further analysis of a stillborn fetus showed that MPXV could infect both the placenta and fetus, causing congenital infection. No additional cases of Clade I MPXV in pregnant women were reported until a new outbreak occurred in South Kivu Province during 2023 and 2024. Eight pregnant women having Clade I MPXV infection were identified, of whom four had either miscarriages or stillbirth, representing a 50% fetal mortality rate. These reports confirm previous data from the DRC that indicate the capability of Clade I MPXV to affect the fetus, causing congenital infection and fetal loss in a high percentage of cases. In this article, we review both past and new data from the DRC on the effects of Clade I MPXV during pregnancy and discuss the association of mpox with fetal loss.

## 1. Introduction

Mpox, formerly monkeypox, is the most life-threatening poxvirus infection since the eradication of smallpox. Mpox is caused by the monkeypox virus (MPXV), an enveloped DNA virus that is a member of the *Orthopoxvirus* genus in the *Poxviridae* family. Mpox was historically concentrated in Central and West African countries where it occurred as sporadic infections and outbreaks in tropical rainforest regions with transmission occurring following animal contact as well as by person-to-person transmission via respiratory droplets, exposure to infectious lesions or fluids, and contaminated materials [1,2,3,4]. MPXV was first recognized as a human pathogen in 1970 following infection of a 9-month-old infant in the Democratic Republic of the Congo (DRC) [5]. After smallpox vaccination was no longer indicated in 1980, and all countries halted vaccine administration by 1984, the prevalence of mpox gradually increased in the endemic African countries, especially the DRC, where it typically infected young, unvaccinated children and, less frequently, their unvaccinated family members [5,6,7,8]. Despite increasing numbers of individual and clusters of cases over the subsequent decades, mpox remained a neglected tropical disease until May 2022 when a multi-country outbreak developed that resulted in confirmed cases in 116 countries, most of which had no direct or immediate epidemiological links to West or Central Africa. This ongoing outbreak has affected primarily (>90%) men who have sex with men, roughly 40% of whom test positive for the human immunodeficiency virus (HIV), and spreads through person-to-person contact via sexual networks [9].

There are two clades, or genetic variants, of MPXV that are endemic in Africa-Clade I (formerly the Central African or Congo Basin clade) and Clade IIa (formerly the West African clade). These MPXV clades have different geographic, epidemiologic, and clinical features. Clade I is associated with more severe disease, has a higher case fatality rate (CFR) averaging 10.6%, and is endemic in the DRC, Central African Republic, Republic of Congo, Sudan, Cameroon and Gabon. Clade I mpox demonstrates smallpox-like features with human-to-human transmission and a high frequency of viremia. Clade IIa has been isolated from outbreaks in Nigeria, Ivory Coast, Sierra Leone, Liberia and the United States, causes milder disease, less viremia and transmissibility, and a lower CFR (1 to 3%) than Clade I MPXV [5,10,11]. The 2022–2024 mpox outbreak was caused by a group of genetic variants which were assigned a new sub-clade, termed Clade IIb [12,13]. 

MPXV has also been shown to infect pregnant women, although much remains unknown regarding how mpox affects mothers, the fetus, placenta and newborn [14,15,16,17,18,19,20]. In this article, we review both past and new data from the DRC on the effects of Clade I MPXV during pregnancy and discuss the association of mpox with fetal loss.

## 2. Mpox in Pregnancy Prior to 2022 

The changing pattern of mpox infections in the DRC from the median age of presentation of 4 years of age in the 1970s to women of reproductive age (21 years) between 2010 and 2019 has been of special concern when dealing with the effects of mpox in pregnancy [5]. Despite the large numbers of infected children and adults in the DRC and elsewhere in Africa, there was scant interest in mpox in pregnancy prior to the 2022 outbreak. Except for a few case reports, there were little data on the spectrum of maternal clinical illness from mpox, the effects of MPXV on the fetus and placenta, and the capability of the virus to exhibit vertical transmission [17,21,22,23,24]. Based upon the few cases of mpox that have been described in pregnant women, different clades of MPXV have demonstrated marked differences in fetal outcomes [17,20]. Clade I, having the highest CFR among all persons infected with MPXV, also has the most severe effects during pregnancy [17]. 

The initial indication that mpox could cause adverse perinatal outcomes was based upon a report from the DRC in 1988 in which a pregnant woman developed clinical evidence of mpox at approximately 24 weeks gestation. Six weeks later she gave birth to a preterm baby with a generalized skin rash that suggested MPXV infection, and the infant died 6 weeks later from malnutrition [22]. It was not until almost 10 years later that confirmatory evidence of MPXV as a lethal pathogen of the fetus was reported from the Kole Human Monkeypox Infection Study conducted in the Sankuru Province of the DRC [21]. A cohort of 222 patients (36% female, 64% male) with symptomatic MPXV infection were enrolled from March 2007 to July 2011 at the General Hospital of Kole in a remote town in the tropical rainforest. Four pregnant women in this study developed mpox that was confirmed by polymerase chain reaction (PCR). The clinical severity of mpox was classified based upon the number of skin lesions as recommended by the WHO as mild (<25 lesions), moderate (25–99 lesions), severe (100–250 lesions) or grave (>250 lesions) [22,25]. Using these criteria, one mother had mild infection (Case #3), two had moderate infection (Cases #1 and #4) and one had severe infection (Case #2). Case 1 had onset of mpox at 6 weeks gestation followed by miscarriage 24 days later. She had moderate mpox disease characterized by fever and 76 skin lesions. Case 2 was a pregnant woman who developed a fever at 6–7 weeks of gestation followed by onset of severe mpox with 1335 skin lesions and miscarriage 2 weeks after onset of fever. Case 3 was a pregnant woman who became febrile at 14 weeks gestation, developed mild clinical mpox disease with 16 skin lesions and had a full-term liveborn infant with no evident infection. Case 4 was the most significant as it was the only case of MPXV infection for which placental and autopsy pathology analysis of perinatal disease is available [17,21,26]. A pregnant woman with malaria became febrile at 18 weeks gestation, developing a moderate MPXV infection with 113 skin lesions. The MPXV viremia increased from 10^2^ to 10^6^ copies/mL when the fetus stopped moving. Transcutaneous amniocentesis was performed which was positive for MPXV. At 21 weeks gestation she gave birth to a stillborn infant having diffuse cutaneous maculopapular lesions involving the chest, head, abdomen, back, shoulders, extremities, palms and soles, which appeared consistent with mpox lesions. Umbilical vein blood tested positive for MPXV using PCR. Autopsy of the stillborn revealed that hepatomegaly, hydrops fetalis and peritoneal effusions were present, with MPXV detected in the skin and liver using both molecular testing and immunohistochemistry. The placenta demonstrated diffuse viral positivity using immunohistochemistry which was predominantly in villous stromal cells (Hofbauer cells) which were increased in number [26,27], thus confirming that intrauterine maternal–fetal transplacental transmission of MPXV can occur [28,29]. Although the numbers were small, these cases suggested that there was a 75% fetal mortality rate for Clade I MPXV infections in pregnant women, adding mpox to the list of TORCH infections that produce congenital disease [17,20,21].

## 3. Mpox in Pregnancy during the 2022–2024 Multi-Nation MPXV Outbreak

The global mpox outbreak that began in May 2022 eventually resulted in 97,745 confirmed cases from 116 countries and 203 fatalities that were propagated almost entirely by person-to-person transmission [30]. Although the overwhelming majority of cases occurred in men who have sex with men, there were at least 58 cases (and probably more) of pregnant women who became infected [28]. Fortunately, there were no confirmed cases of fetal infection or intrauterine transmission reported [15,17,20,28]. The 2022–2024 MPXV outbreak was found to result from an offshoot of the Clade II (West African) strain that possessed novel mutations and was classified as a new strain, Clade IIb [31]. The lack of perinatal morbidity from Clade IIb might be explained in several ways: with infected pregnant women composing a very small percentage of the total cases, the outbreak having a <0.1% overall case fatality rate from MPXV Clade IIb infection, and Clade IIb causing a milder form of disease than the MPXV Clade I or IIa variants [20,28].

## 4. The 2023–2024 Mpox Outbreak in South Kivu, DRC

The DRC is the most affected country by MPXV in the world, having reported cases of mpox continuously for the previous 50 years. Following the cessation of smallpox vaccination, the incidence of MPXV infections have increased. Although the exact numbers of mpox cases in the DRC are difficult to determine with accuracy, from 1981 to 1986 the WHO reported 338 confirmed cases and 33 fatalities, a CFR of 9.8%. Between 1991 and 1998, there were 511 cases of mpox with some estimating that there were more than 2000 suspected cases, and between January and September 2020, there were 4594 suspected cases [17].

Since the beginning of 2023 there has been a surge of mpox infections in remote parts of the DRC where the disease had not been previously identified [32]. A new outbreak of mpox was reported in the South Kivu Province in September 2023 [32,33], which has since spread into the adjacent North Kivu Province [34,35]. Sexual transmission of Clade I MPXV in the DRC was initially described from a small cluster of infections occurring in Kwango Province in April 2023 [36], after which additional cases were reported in September 2023 following sexual contact with a known mpox case in Kamituga health zone, South Kivu province. This outbreak was caused by an MPXV strain with a novel Clade I lineage carrying predominantly *APOBEC3*-type mutations, indicating adaptation of MPXV to circulation among humans, that emerged in mid-September 2023 and that has resulted in continuous human-to-human transmission. This new strain is missing a target sequence recognized by nucleic acid probes and primers of a frequently used Clade I-specific PCR test [32,33]. As of May 26 2024, there have been a total of 7851 mpox cases resulting in 384 deaths (CFR 4.9%) reported from 177/519 health zones across 22/26 provinces with most infections acquired through heterosexual contacts including commercial sex workers and their contacts [34]. The median age of affected persons in this outbreak is 21 years, with most (59%) between 15 and 29 years of age and greater than 50 percent of cases occurring in women [37]. This represents a markedly different epidemiology than the 2022–2024 multi-nation MPXV epidemic in which 98 percent or more of affected persons were gay or bisexual men [38].

To better understand the epidemiology of this outbreak, Masirika and colleagues enrolled 371 patients hospitalized at the Kamituga Hospital with confirmed, probable or suspected mpox during the period 29 September 2023 to 21 April 2024 [32]. The Kamituga health zone, where this hospital is located, is the epicenter of the mpox outbreak and the only health zone in South Kivu that has continuously identified mpox cases since the start of the outbreak. Kamituga is the largest gold mining city in South Kivu and has a large number of bars that support commercial sex workers. Significantly, in this cohort there were eight pregnant women admitted to the hospital, of whom four had fetal losses—a fetal mortality rate of 50 percent. Two women who had spontaneous abortions developed clinically severe mpox symptoms during the first quarter (first 10 weeks) of their pregnancy with confirmed MPXV infection by PCR and were negative for HIV. A third pregnant woman, positive for HIV, developed severe mpox symptoms during the second quarter (between 10 and 20 weeks gestation) and had a spontaneous abortion confirmed by ultrasound. At the time of delivery, the macerated fetus was found to have skin rashes similar to those of mpox. The fourth woman became severely ill with mpox requiring hospitalization for 21 days. She had a spontaneous abortion during the first quarter of pregnancy and tested negative for HIV. All of the pregnant women had exposure to persons infected with MPXV through bar visits. There were no autopsies performed on any of the four fetuses and the placentas were not examined. 

## 5. Discussion

The original description of a 75 percent rate of fetal loss among pregnant women with mpox from the 2007–2011 Kole Monkeypox Study was highly suggestive of the lethal potential of Clade I MPXV during pregnancy [21]. This study also provided the first autopsy-confirmed case of fetal infection by MPXV, as well as documenting infection of the placenta in which the virus diffusely involved the stromal cells of the chorionic villi and confirming transplacental transmission [17,21,26,27,28]. However, the new data describing intrauterine fetal death occurring in 50 percent of pregnant women having mpox in the Kamituga Health zone study are of great significance in providing confirmatory evidence of the potential effect of MPXV as an agent of congenital infection, miscarriage and stillbirth. Although there are many viral agents that can have adverse effects on the developing fetus, there are very few that produce fetal demise in the range of 50 to 75% of infected mothers. This level of mortality may even be high when compared with the effect of variola virus on the fetus prior to its eradication in 1980. Because smallpox was responsible for a high level of maternal mortality, it is now difficult to determine the effects of the variola virus on the fetus [17]. Cases of congenital infection and perinatal deaths from smallpox were reported, but the frequency of these outcomes among mothers having variola virus infection is unknown [17].

Another interesting finding is that all three of the mothers having mpox and miscarriage or fetal demise in the Kole Monkeypox Study were in the first or early second trimester of pregnancy, while the four infected mothers in the Kamituga Hospital study who had adverse fetal outcomes were in the first or second quarter of pregnancy [21,27,32]. While the number of cases is too small to draw any meaningful conclusions, it could be possible that the developing fetus and its placenta are most sensitive to MPXV early in gestation as occurs in some other viral infections such as rubella and cytomegalovirus.

Among pregnant women with Clade I mpox, the role of co-infections with adverse perinatal outcomes is unknown. However, among the seven mothers in the two studies having fetal losses due to MPXV, one woman had concurrent HIV infection [31] and another had malaria [21]. In the DRC, there is a high prevalence of HIV in women [39], which is important because persons living with HIV who develop mpox have had worse clinical outcomes than non-HIV-infected individuals in several studies [40]. We now have definitive evidence from two separate prospective studies that were conducted many years apart in differing regions of the DRC showing that Clade I MPXV produces high rates of fetal loss among pregnant women with mpox [17,20,21,27,28,32,33]. Although this represents a small number of cases from which to draw any definitive conclusions, the affected pregnancies were all from two closely supervised population-based studies that prospectively enrolled persons of all ages and genders having symptomatic mpox, regardless off severity. Thus, it would appear that these cases are likely typical of the spectrum of potential effects of MPXV on the developing fetus in infected mothers. It would be important to evaluate the perinatal outcomes from greater numbers of pregnant women with Clade I MPXV infection to confirm this high rate of fetal loss. The high prevalence of mpox occurring in women of reproductive age during the 2022–2024 South Kivu mpox outbreak is worrisome for the development of additional cases of vertical MPXV transmission and adverse perinatal outcomes. In addition, in any future cases of mpox associated with fetal loss or vertical infection, it is important that pathological evaluation of fetal tissues including the placenta be performed using both routine as well as antibody- and nucleic acid-based methods to further understand the pathophysiology of the effect of mpox on adverse pregnancy outcomes. 

Now, the medical and public health communities should prioritize potential strategies for prevention including early case identification and diagnosis, vaccination and potential therapy. Although cases of mpox that have been occurring outside of Africa have been due to the milder Clade II variants, it should not be assumed that Clade I MPXV will not travel outside of the endemic areas. The recent increase in cases of Clade I MPXV in the DRC, including the emergence of a novel viral strain, creates a heightened risk for spread of the virus globally if its transmission is not controlled. MPXV, together with variola major (smallpox) virus, is on the list of biological agents that represent a severe threat to human health from the United States Public Health Security and Bioterrorism Preparedness and Response Act [41]. Mpox is too dangerous a disease to remain neglected any longer, especially when it develops during pregnancy.

## Data Availability

Not applicable.
